# A Case of Severe Kratom Addiction Contributing to a Suicide Attempt

**DOI:** 10.7759/cureus.29698

**Published:** 2022-09-28

**Authors:** Austin G Settle, Chong Yang

**Affiliations:** 1 Psychiatry, Touro University California, Vallejo, USA; 2 Forensic Psychiatry, California Department of State Hospitals, Napa, USA

**Keywords:** suicide attempt, kratom addiction, harm reduction, opioid use disorders, withdrawal, addiction, kratom

## Abstract

Kratom is a plant product native to Southeast Asia that is surging in popularity in the United States as consumers look for natural remedies for ailments like chronic pain, anxiety, and even opioid addiction. Kratom's risks are largely unknown, and the market is poorly regulated. Americans typically get advice from online forums and purchase powder formulations from tobacco shops or obscure websites. These risks are highlighted in this case of a 38-year-old woman with a history of major depressive disorder and opioid use disorder who switched from Suboxone to kratom and became addicted, eventually consuming large quantities per day. Amplified by other stressors, she reached a breaking point and overdosed on her regular medications for depression. At the psychiatric hospital, she exhibited classic kratom withdrawal symptoms, including vague abdominal pain and restlessness. After eight days of treatment, her symptoms eventually abated, and she was discharged on Suboxone.

## Introduction

Kratom is a tea leaf extract derived from the plant *Mitragyna speciosa* that is native to Malaysia, Myanmar, and Thailand. In Southeast Asia, its plant leaves have been utilized for centuries for various applications. Kratom provides stimulating effects at low doses and farmers use it to be more productive and stave off fatigue [1]. It was also historically consumed to provide relief for other ailments, including fever, cough, diarrhea, and even hypertension. Its contemporary forms vary widely in its native regions where it is still commonly chewed or boiled in tea. In the United States, it is typically formulated into powders or marketed as a concentrated energy shot in convenience stores like gas stations and tobacco shops. These formulations are often mixed into drinks for ease of consumption [2].

An analysis of the 2019 National Survey on Drug Use and Health estimated that the proportion of Americans over the age of 11 who used kratom in the past year is about two million people [3]. The target audience includes middle-income, young-to-middle-aged adults seeking out remedies for pain or mental health conditions like anxiety [4]. Pain relief is the most commonly cited reason for the consumption of kratom [5]. The results of national surveys also indicate that a substantial fraction of users take kratom primarily as a replacement for prescription or over-the-counter medications [6]. In a cross-sectional survey of over 8,000 members of the American Kratom Association (AKA), around 80% of users claimed they experienced decreased pain, increased energy, and improved mood. Approximately half of them used kratom to stop or reduce opioid use [4]. The harm reduction potential of kratom has been supported by research such as a study by Yue et al. in which rodents provided with regular mitragynine were less likely to self-administer heroin compared to controls [7].

The understanding of kratom’s pharmacology including its mechanism of action is limited. However, two active components, mitragynine and 7-hydroxymitragynine (7-HMG), have been identified to play an active role. These compounds are indole alkaloids that act as partial agonists of the mu-opioid receptor. They also fail to recruit beta-arrestin 2, a protein associated with many of the dangerous effects of opioids like central nervous system depression, constipation, and dependence [5]. Kratom, however, still possesses significant side effects and addictive properties. A large survey by the AKA revealed that the most common side effects were nausea, constipation, and other gastrointestinal issues that presented at higher or more frequent dosing [4]. Experiments on mice suggest that regular mitragynine administration is associated with withdrawal symptoms like anxiety and cognitive impairment [8]. Commonly reported withdrawal symptoms mirror those of opioids and include anxiety, nausea, vomiting, diarrhea, abdominal cramps, and muscle cramps [9]. Studies have revealed that mitragynine has a diverse range of actions that may induce pain suppression through a proposed affinity to dopamine and serotonin receptors. Matsumoto et al. discovered that mice provided with supraspinal mitragynine injections revealed antinociceptive activity involving both descending noradrenergic and serotonergic systems [10].

Kratom use in America is a divisive issue that has only been exacerbated by rising demand (and, naturally, kratom-related toxic events) combined with a poor scientific understanding of its risks. The Centers for Disease Control and Prevention reported that, from 2010 to 2015, there were 660 kratom-related calls to US poison control centers, with 428 noted as isolated kratom use. The most commonly reported adverse effects were nausea, vomiting, tremor, diaphoresis, tachycardia, and hypertension [9]. Unfortunately, these calls have been increasing as 2/3 of the over 1800 calls related to kratom ingestion that were received from 2011 to 2017 occurred in the last two years of that period [1]. Kratom is currently considered a “Drug and Chemical of Concern” by the Drug Enforcement Administration (DEA). The Food and Drug Administration issued a public health warning about the substance in 2017 and at that time reported 36 deaths due to illicit use of kratom-containing products [9]. In the following case report, we present a patient with opioid use disorder who consumed kratom as a substitute for buprenorphine/naloxone and experienced an episode of acute withdrawal.

## Case presentation

Psychiatric history

Mrs. K is a 38-year-old woman with past psychiatric history of major depressive disorder and opioid use disorder (in remission with her last heroin use eight years ago) who presented to the emergency department (ED) after an intentional overdose suicide attempt. Her history indicated that she consumed unknown quantities of quetiapine and duloxetine. Her alcohol level was 125. She described mounting familial stress, dysphoric mood, and low motivation that caused her to isolate herself in her apartment for several weeks until she finally acted on an impulse to take a large handful of her pills. In the ED, she was lethargic and moderately hypotensive with mild lactic acidosis. Her urine drug screen was positive only for cannabis. Other than lingering hypotension, her clinical presentation improved rapidly. Throughout her hospitalization, she did not present with abnormal respiratory rate, heart rate, or heart rhythm. Scores on Clinical Institute Withdrawal Assessment for Alcohol and Clinical Opiate Withdrawal scales were in the mild category, or less than 10 and 12, respectively. Overnight, nursing noted fluctuations between a somnolent and irritable/anxious mood in which she cried and complained of anxiety, nausea, and body pains, specifically her entire abdomen. She admitted to drinking the night before, claiming it was more than usual and denying any history of alcohol abuse. Her labs and imaging were normal, and no abdominal tenderness upon palpation was found. She was subsequently transferred to the psychiatric hospital.

In the psychiatric hospital, Mrs. K shared her history of substance use including recently consuming large amounts of kratom regularly. She stated that she was a regular heroin user throughout her 20s but has not used it for eight years with the aid of buprenorphine/naloxone. She first started using kratom eight months ago after relocating from another state and losing services from her previous Suboxone clinic. She reported that around that time, her mood was normal and had been well-controlled by her regular medications. Although, she still felt high baseline anxiety most days. Initially, she used kratom as a Suboxone replacement upon recommendation from a friend, but eventually, she found that it provided sedation to help her with anxiety and even a mild sense of euphoria. She obtained it from smoke shops. She started with one or two tablespoon(s) of kratom per day and gradually increased her consumption, driven by increasing tolerance and cravings. Eventually, she consumed up to five to six tablespoons per day and felt that she needed it to function and would have agitation, anxiety, shakes, and spasms of her extremities without it. Acquiring this massive quantity of kratom was costing her as much as $75 per week. Around this period, she began isolating herself in her bedroom, not socializing with anyone. She admitted that it had taken over her life and worsened her mood. It caused her to live in fear of her anxiety returning, adding to her ongoing stress of loneliness in a new city and acrimony with her family.

With regards to the Diagnostic and Statistical Manual of Mental Disorders, Fifth Edition (DSM-V) diagnostic criteria for substance use disorder, Mrs. K satisfied over two criteria over the past 12 months, which is enough to qualify for kratom use disorder [11]. She had cravings to use the substance, social activities given up because of use, a great deal of time spent obtaining and using the substance, taking larger amounts or over a longer time than was originally intended, tolerance, and withdrawal. By fulfilling six points of the criteria, the severity of her disorder can be specified as severe [11].

Treatment

Mrs. K did not endorse active suicidal ideation throughout her psychiatric hospitalization. However, she continued to complain of depressed mood, anxiety, poor sleep, abdominal pain, occasional diarrhea, and restlessness, including intermittent leg twitching and handshaking. She presented with akathisia as she demonstrated an inability to sit still. She was argumentative with staff and continued to ask for benzodiazepines. On her second day, she reported craving kratom and revealed the full extent of her kratom use. Her home medications (quetiapine 25 mg at night and duloxetine 30 mg per day) were restarted in the hospital, and she was provided melatonin for sleep. She denied cravings for other opioids. Buprenorphine was added on her fifth day after reaching an agreement to return to buprenorphine/naloxone for maintenance once discharged.

Response to treatment

Minimal improvement was noticed in Mrs. K’s first two hospital days, but afterward, she demonstrated consistent gradual improvements in her mood, anxiety, restlessness, and agitation. She no longer complained about poor sleep by day six. She was able to sit calmly and displayed increased participation in group therapy sessions. She had occasional episodes of diarrhea that improved over the course of her hospitalization. Similarly, she reported decreased abdominal pain and the resolution of any residual abdominal pain upon discharge. She did not feel the need to follow up with a gastroenterologist despite our recommendation. She decided to return to buprenorphine/naloxone for opioid maintenance after her fifth day once she gained awareness of the severity of her kratom addiction; she wanted the security of medication-assisted treatment despite not craving heroin for years. She was scheduled for an appointment at a local Suboxone clinic. She was discharged with a prescription for buprenorphine 2 mg/naloxone 0.5 mg three times a day, which was her preference for dosing. She denied any desire to resume kratom. She claimed the cravings had subsided and noted that she could not afford it. Retrospectively, she admitted that she enjoyed its sedating effects. She also stated, "I need to treat my underlying anxiety, and kratom does not do that. And I do think it’s addictive. I would never want my child taking it.”

## Discussion

Considering Mrs. K's recent history of opioid abstinence, regular, large-quantity kratom consumption, and satisfaction of DSM-V substance use disorder criteria, a compelling case can be made that she suffered from kratom use disorder and eventually withdrawal. While it lacks the severe opioid toxicity of respiratory depression, it retains classic opioid withdrawal symptoms like anxiety, nausea, vomiting, diarrhea, abdominal cramps, muscle spasms, and insomnia [9]. During her hospitalization, Mrs. K experienced almost all of these symptoms except for vomiting. Her symptoms did not begin improving until after two days into her hospitalization. Her abdominal pain was reported as dull, diffuse, and intermittent with no associated tenderness to palpation. She mentioned that it made her feel weak. She was even briefly sent back to the ED after her first day in the psychiatric hospital due to concerns about her pain, but, again, no abnormalities were detected. Her anxiety is difficult to attribute solely to kratom withdrawal as it could have also been due to her stressful situation. This complex interplay of factors raises the question of how her escalating kratom use might have affected her anxiety or mood, as her severe depressive episode and suicidal ideation arose despite her reporting that her medication regimen had been controlling her depression effectively for years. She had no clear acute stressors other than feeling shut out by her family and difficulty acclimatizing to a new state. While it is impossible to delineate the causes and effects, in this case, the interplay of kratom dependence and then withdrawal with her depression and suicide attempt are intriguing.

Mrs. K was taking extremely high doses of kratom, which contributed significantly to her withdrawal symptoms. This may have even delayed her recovery as kratom withdrawal typically resolves in one to three days [12]. The American Kratom Association’s 8,000-user survey revealed that a dose of five grams and above was considered a high dose. Toxicity, especially nausea and constipation, was much more likely to occur when doses exceeded eight grams or when the frequency was over 22 doses per week [4]. Mrs. K was taking five to six tablespoons per day. According to dosing estimates proposed by Shah et al., this consumption equates to about 35 to 42 grams of the specific type of kratom powder [13]. She typically drank a mixture of between two to three tablespoons of kratom powder and apple juice twice a day. Sanderson and Rowe found that doses over 15 g may mimic an opioid toxidrome [14]. Clearly, Mrs. K developed tolerance after months of consistent use and increasing dosages, as she did not report any recent history of opioid toxicity symptoms. Multiple studies have shown that kratom tolerance and physical dependence develop over time in a manner similar to coffee or milder opioids [2]. Just how much, if at all, her dependence, tolerance, and near-constant consumption contributed to her depressive episode is unclear. No studies have shown any kind of relationship between depression and long-term use, but her extreme daily intake is also fairly unprecedented. Consistent high doses have mostly been linked to gastrointestinal symptoms such as constipation and nausea [2].

Treating kratom addiction and withdrawal is up to individual providers as no evidence-based consensus has been established. For the treatment of kratom addiction, replacement therapy with buprenorphine is a reasonable approach, and reports have shown its effectiveness for kratom replacement [1]. Research shows that kratom withdrawal typically resolves on its own, but this finding is limited to self-reported responses [12]. Case reports have demonstrated successful treatment with various medications used to treat opioid withdrawal such as buprenorphine, clonidine, and even morphine for a mother and neonate [9]. During Mrs. K’s hospitalization, our approach was to manage her withdrawal without adding any specific medications for her symptoms. This decision is partly due to her unknown consumption of other substances and an intent to refrain from providing benzodiazepines to someone with a history of substance abuse. Additionally, after persistent hypotension in the emergency department, her vital signs were stable throughout her hospitalization. It required eight total days of hospitalization to achieve near-complete resolution of her insomnia, anxiety, and abdominal pain. Her withdrawal may have been eased and expedited by starting buprenorphine earlier.

Our patient asserted that kratom was equally effective at preventing opioid cravings as Suboxone. Kratom’s harm must be established before considering its adoption for harm reduction. One recent survey of over 6,400 kratom users elucidated its harm reduction benefits as around 75% of users answered “no” to the question “can you get high from using kratom?”, and 69% of users answered that they would seek out opioids as a replacement if kratom became illegal [6]. Research on Malaysians who replaced heroin with kratom has shown significant decreases in the prevalence and severity of self-reported adverse effects such as respiratory depression, constipation, physical pain, insomnia, depression, and craving [15]. The long-term risks of kratom use are unclear. Case studies suggest that chronic use may cause cognitive impairment, seizures, and other neurological impairments [1]. A study on mice revealed that chronic administration of mitragynine led to decreased performance on some cognitive tests such as avoidance learning and object recognition [8]. The poorly regulated market has led to the proliferation of adulterated kratom products that likely pose more risk than their pure form. For example, a mix called Krypton, which contains a metabolite of the opioid tramadol, has been directly attributed to nine deaths [6]. According to Swogger et al., a Food and Drug Administration report that cited 44 deaths due to kratom failed to consider that almost all cited deaths involved kratom products with synthetic additives and/or the co-ingestion of substances such as heroin and synthetic opioids [2]. For now, users of kratom need guidance from physicians and public health authorities to avoid the known risks including its addiction potential. The case of Mrs. K supports this dire need.

## Conclusions

Kratom has the potential as a legitimate replacement for opioids in an appropriate setting, but it is not entirely free of side effects. It is clear that kratom still has addictive properties and can upend someone’s life with the all too familiar bane of dependence and withdrawal, as seen in the case of Mrs. K. Tolerance to large quantities can be developed, which leads to significant financial drain and perhaps an even worse toll on mental health over time. Kratom withdrawal can be quite debilitating as well, and it can mimic opioid withdrawal. It is critical that providers ask about kratom use history in cases with similar presentations to guide their treatment. Our patient’s recovery might have been alleviated and expedited by the early addition of clonidine or buprenorphine. Despite kratom’s dangers, many users report satisfaction with improved mood and chronic pain symptoms. Its use in America is growing rapidly, and misinformation and poorly manufactured products are spreading. Additional research is needed to fully understand its benefits and risks, and careful guidance must be provided from providers and public health authorities to mitigate cases like Mrs. K.
